# SNP Array Analysis Reveals Novel Genomic Abnormalities Including Copy Neutral Loss of Heterozygosity in Anaplastic Oligodendrogliomas

**DOI:** 10.1371/journal.pone.0045950

**Published:** 2012-10-10

**Authors:** Ahmed Idbaih, François Ducray, Caroline Dehais, Célia Courdy, Catherine Carpentier, Simon de Bernard, Emmanuelle Uro-Coste, Karima Mokhtari, Anne Jouvet, Jérôme Honnorat, Olivier Chinot, Carole Ramirez, Patrick Beauchesne, Alexandra Benouaich-Amiel, Joël Godard, Sandrine Eimer, Fabrice Parker, Emmanuelle Lechapt-Zalcman, Philippe Colin, Delphine Loussouarn, Thierry Faillot, Phong Dam-Hieu, Selma Elouadhani-Hamdi, Luc Bauchet, Olivier Langlois, Caroline Le Guerinel, Denys Fontaine, Elodie Vauleon, Philippe Menei, Marie Janette Motsuo Fotso, Christine Desenclos, Pierre Verelle, François Ghiringhelli, Georges Noel, François Labrousse, Antoine Carpentier, Frédéric Dhermain, Jean-Yves Delattre, Dominique Figarella-Branger, POLA Network

**Affiliations:** 1 Université Pierre et Marie Curie-Paris 6, Centre de Recherche de l'Institut du Cerveau et de la Moelle Epinière (CRICM), UMRS 975, Paris, France; 2 Inserm U 975, Paris, France; 3 CNRS, UMR 7225, Paris, France; 4 AP-HP, Groupe Hospitalier Pitié-Salpêtrière, Service de neurologie 2-Mazarin, Paris, France; 5 INSERM U1028, CNRS UMR5292, Service de Neuro-oncologie, Hôpital neurologique, Hospices civils de Lyon, Lyon Neuroscience Research Center, Neuro-Oncology and Neuro-Inflammation Team, Lyon, France; 6 AP-HM, Hôpital de la Timone, Service d'anatomie pathologique et de neuropathologie, Marseille, France; 7 Université de la Méditerranée, Aix-Marseille, Faculté de Médecine La Timone, CRO2, UMR 911 Marseille, France; 8 AltraBio, Lyon, France; 9 Service d'anatomie pathologique et histologie-cytologie, Hôpital de Rangueil, CHU de Toulouse, Toulouse, France; 10 AP-HP, Groupe Hospitalier Pitié-Salpêtrière, Service de neuropathologie Raymond Escourolle, Paris, France; 11 Service d'anatomo-pathologie, Groupement hospitalier Est, Service de neurologie B, Hôpital Pierre Wertheimer, Hospices Civils de Lyon, Lyon, France; 12 Unité de Neuro-oncologie, Hôpital de la Timone, Marseille, France; 13 Clinique de neurochirurgie, CHU de Lille, Lille, France; 14 Service de neurologie, CHU de Nancy, Nancy, France; 15 Service de neurologie, Hôpital Purpan, CHU Toulouse, Toulouse, France; 16 Service de neurochirurgie, Hôpital Jean Minjoz, Besançon, France; 17 Service de pathologie-neuropathologie, Hôpital Pellegrin, CHU Bordeaux, Bordeaux, France; 18 Service de neurochirurgie, Hôpital Bicêtre, Le Kremlin-Bicêtre, France; 19 Service d'anatomie pathologique, Hôpital de la Côte de Nacre, CHU de Caen, Caen, France; 20 Service d'oncologie-radiothérapie, Clinique de Courlancy, Rheims, France; 21 Service d'Anatomie Pathologique B, Hôpital G&R Laënnec, Nantes, France; 22 Service neurochirurgie, Hôpital Beaujon, Clichy, France; 23 Service neurochirurgie, Hôpital de la cavale blanche, Brest, France; 24 Service de neurochirurgie, Hôpital Lariboisière, Paris, France; 25 Service neurochirurgie, Hôpital Gui de Chaulliac, Montpellier, France; 26 Service de neurochirurgie, CHU Charles Nicolle, Rouen, France; 27 Service de neurochirurgie, Hôpital Henri Mondor, Créteil, France; 28 Service de neurochirurgie, Hôpital Pasteur, Nice, France; 29 Département d'oncologie médicale, Centre Eugène Marquis, Rennes, France; 30 Service de neurochirurgie, CHU Angers, Angers, France; 31 Service de neurochirurgie, Hôpital Nord, CHU Saint-Étienne, Saint-Priest en Jarez, France; 32 Service de neurochirurgie, Hôpital Nord, CHU Amiens, Amiens, France; 33 Département de radiothérapie, Centre Jean Perrin, Clermont-Ferrand, France; 34 Département d'oncologie médicale, Centre Georges-François Leclerc, Dijon, France; 35 Département de radiothérapie, Centre Paul Strauss, Strasbourg, France; 36 Service d'anatomopathologie, CHU Dupuytren, Limoges, France; 37 Service de neurologie, Hôpital Avicenne, Bobigny, France; 38 Service de radiothérapie-radiophysique, Institut Gustave Roussy, Villejuif, France; Deutsches Krebsforschungszentrum, Germany

## Abstract

Anaplastic oligodendrogliomas (AOD) are rare glial tumors in adults with relative homogeneous clinical, radiological and histological features at the time of diagnosis but dramatically various clinical courses. Studies have identified several molecular abnormalities with clinical or biological relevance to AOD (*e.g.* t(1;19)(q10;p10), *IDH1*, *IDH2*, *CIC* and *FUBP1* mutations).

To better characterize the clinical and biological behavior of this tumor type, the creation of a national multicentric network, named “*Prise en charge des OLigodendrogliomes Anaplasiques* (POLA),” has been supported by the *Institut National du Cancer* (InCA). Newly diagnosed and centrally validated AOD patients and their related biological material (tumor and blood samples) were prospectively included in the POLA clinical database and tissue bank, respectively.

At the molecular level, we have conducted a high-resolution single nucleotide polymorphism array analysis, which included 83 patients. Despite a careful central pathological review, AOD have been found to exhibit heterogeneous genomic features. A total of 82% of the tumors exhibited a 1p/19q-co-deletion, while 18% harbor a distinct chromosome pattern. Novel focal abnormalities, including homozygously deleted, amplified and disrupted regions, have been identified. Recurring copy neutral losses of heterozygosity (CNLOH) inducing the modulation of gene expression have also been discovered. CNLOH in the *CDKN2A* locus was associated with protein silencing in 1/3 of the cases. In addition, *FUBP1* homozygous deletion was detected in one case suggesting a putative tumor suppressor role of *FUBP1* in AOD.

Our study showed that the genomic and pathological analyses of AOD are synergistic in detecting relevant clinical and biological subgroups of AOD.

## Introduction

Anaplastic oligodendrogliomas (AOD) are rare primary brain tumors that account for approximately 10% of all gliomas [1], [2]. AODs are a heterogeneous subgroup of tumors with distinct biological features and clinical behavior despite their homogeneous morphological appearance when viewed under a microscope, including oligodendrocyte-type cells that form honey combs and anaplastic features with a high cell density, cytonuclear atypia, mitosis, vascular proliferation and, in some cases, necrosis [3].

Despite similar treatments and histologic features, AOD patients can have dramatically different outcomes: (i) ∼25% of the patients die within 18 months of diagnosis, similar to glioblastoma patients and (ii) ∼25% survive more than 8 years, similar to low-grade glioma patients [4], [5]. Therefore, the AOD group encompasses several entities in terms of its clinical and biological characteristics.

Genomic studies have shown an ability to identify molecular abnormalities in AOD tumors, which are necessary for a better understanding of molecular oligodendrogliomagenesis and for use in clinical practice as relevant biomarkers. The co-deletion of the chromosome arms 1p/19q, mediating an unbalanced reciprocal translocation expressed as t(1;19)(q10;p10), and the *IDH1* and *IDH2* (isocitrate dehydrogenase 1 and 2) mutations have been shown to be recurring in AOD and to be associated with a better outcome [6]–[11]. On the other hand, gene amplification, no matter the targeted gene, is associated with a poor prognosis [12]. More recently, recurring point mutations targeting *CIC* (capicua homolog) and *FUBP1* (Far Upstream Element [FUSE] Binding Protein 1) have been discovered in the majority of AOD cases, further specifying the oncogenetics of AOD [13]; however, the clinical-biological value of the latter genetic abnormalities has only begun to be investigated [14], [15].

The rare nature of AOD requires the use of collaborative multicentric works. To improve the clinical, biological and translational research focused on AOD patients, the *French Institut National du Cancer* (InCA) has supported the creation of a national network named “*Prise en charge des OLigodendrogliomes Anaplasiques* (POLA),” which is dedicated to the harmonization of the clinical management of AOD patients and the development of translational research in AOD.

In this setting, the present study has been conducted by the POLA network in order to identify novel genomic abnormalities in AOD, using high-resolution single nucleotide polymorphism arrays (SNP array), and to correlate the genomic pattern with the *IDH1*/*2* mutations.

## Materials and Methods

### Materials

Eighty-three patients with a centrally reviewed diagnosis of brain AOD were prospectively included in the present study. For all of the individuals, frozen and formalin-fixed paraffin-embedded (FFPE) tumor tissues were available for the genetic, pathological and immunohistochemical investigations. A blood sample was collected and stored at −20°C until use for research purposes, before any anti-tumor treatment was started, as recommended by POLA network policy. The patients included prospectively in the POLA network have provided their written consent for the clinical data collection and genetic analysis according to the national and the POLA network policies.

### Methods

#### Pathological review

After the initial local diagnosis of AOD, the pathological slides were centrally reviewed by Dr. DFB (or Dr. KM for the patients clinically managed in the city of Marseille) and were included in the prospective POLA network if they met the pathological inclusion criteria of AOD, as defined by the World Health Organization classification of brain tumors [3]. All cases were also reviewed by a panel of four neuropathologists D-FB, K-M, A-J and E-UC to be definitely included in the present series.

#### DNA extraction

DNA was extracted from frozen tumor samples using the iPrep ChargeSwitch® Forensic Kit, according to the manufacturer's recommendations. Blood DNA was extracted using conventional method. The DNA was quantified and qualified using a NanoVue spectrophotometer and gel electrophoresis. A volume of 1.5 µg of DNA was outsourced to Integragen Company (Paris, France) for the SNP array experiments.

#### SNP array procedures

As mentioned above, the SNP array experiments were outsourced to Integragen. Two types of platforms were used, HumanCNV370-Quad and Human610-Quad from Illumina. Because the molecular abnormalities were included in the medical management of the patients (*e.g.*, non-1p/19q-co-deleted patients were included in the EORTC 26053-22054 trial if they were eligible elsewhere; [16]), the tumor DNA was run prospectively in order to obtain the genomic profile within ten days of the tumor resection. One microgram of tumor DNA was moved to the higher resolution platform after its implementation, as a service by Integragen®.

#### Real-time polymerase chain reaction (PCR) testing of CDKN2A and EGFR

The *CDKN2A* (Hs02738179_cn) homozygous deletions and *EGFR* (TaqMan® EGFR probe) high-level amplifications were validated using the MGB-based TaqMan® Copy Number Assay (Applied Biosystems), according to the manufacturer's recommendations. RNase P (RNase P Kit, Applied Biosystems, reference: 4403326) was used as the control for assessing the normal copy number status. Briefly, all assays were run in duplicate on a LightCycler®480 Multiwell Plate 96 in a 20 µL reaction volume (10 µL of LightCycler®480 Probes Master Mix, 1 µL of TaqMan® Copy Number Assay, 1 µL of TaqMan® Copy Number Reference Assay and 5 ng of genomic tumor DNA) with the following PCR conditions: initial activation step at 95°C for 10 min followed by 50 cycles of 95°C for 15 s and 60°C for 1 min. The 2^−ΔΔCt^ method was used to obtain the gene's copy number status.

#### IDH1 and IDH2 mutational status

*IDH1* codon 132 and *IDH2* codon 172 were sequenced using the Sanger method with the following primers: IDH1-Forward: TGTGTTGAGATGGACGCCTATTTG; IDH1-Reverse: TGCCACCAACGACCAAGTC; IDH2-Forward: GCCCGGTCTGCCACAAAGTC and IDH2-Reverse: TTGGCAGACTCCAGAGCCCA, as previously reported [17].

#### CDKN2A immunochemistry

A 4 µm thick section of formalin-fixed paraffin-embedded blocks was immunostained using the monoclonal antibody anti-CDKN2A (Clone E6H4 from CINTEC, prediluted) after antigen retrieval, to assess the CDKN2A expression. A Ventana Benchmark XT was instrumental in performing this technique. No immunoreactivity was scored in the CDKN2A protein silencing.

#### Chromosome 9p microsatellite analysis

The blood and tumor DNA were investigated for loss of heterozygosity (LOH) on chromosome 9p using the following polymorphic markers: D9S1684, D9S171 and D9S1121. The forward primer was labeled with the Fam (D9S1684 and D9S1121) or Ned (D9S171) dyes (Life Technologies™). The primer sequences are available upon request. The samples were run on an automatic sequencer and analyzed with the Gene Scan program (Abi-prism, Perkin Elmer).

#### Statistical analyses

The total and allele-specific copy numbers were computed for each sample using the crlmm algorithm [18], [19]. The correction of the total copy number waves was based on the GC-content of the probes and targeted DNA regions. The total copy numbers from the tumor samples were normalized using the blood sample from the same patient, when available, or with the median signal of all the blood samples processed using the same Illumina platform. The B-allele frequencies from patients with two samples processed using the same Illumina array type were corrected using the TumorBoost algorithm [20].

Segmentation, segment categorization and tumor purity estimation were performed using a slightly modified version of genoCN [21]. In the original algorithm, constraints were placed on the lower and upper values of the estimated model parameters. These bounds were not allowed to evolve during the optimization process. For samples of lower tumor purity, this can adversely affect the proper estimation of the parameters. In the modified version of the algorithm, these limits are updated at each iteration to account for their credible values based on the current estimation of sample purity. All the modifications brought to the version 1.8.0 of genoCN (http://www.bioconductor.org/packages/2.10/bioc/html/genoCN.html) are reported in supporting file. Only segments with at least 10 SNPs were retained.

For each segment, the association of its loss (LOH) or gain with each phenotypic variable was estimated using Fisher's exact test for the factors or by the t-test for the quantitative variables. The false discovery rate was controlled using the Benjamini and Hochberg control of p-values [22].

Age and Karnofsky performance status (KPS) were compared using a two-sample t-test. Sex distribution among groups was compared using Chi-squared test with Yates' correction. Progression free survival and overall survival curves were drawn using the Kaplan-Meier method and compared using a log-rank test. A p-value<0.05 was considered as significant.

## Results

### Population characteristics and pathological features of the tumors

Eighty-three patients were prospectively included in the present study: 48 males and 35 females (sex ratio: m/f = 1.4). The median age at diagnosis was 49.9 years (range: 23.1–78.4).

All tumors were validated as AOD after a central pathological review.

### Genomic pattern and IDH mutational status

**Figure 1:**
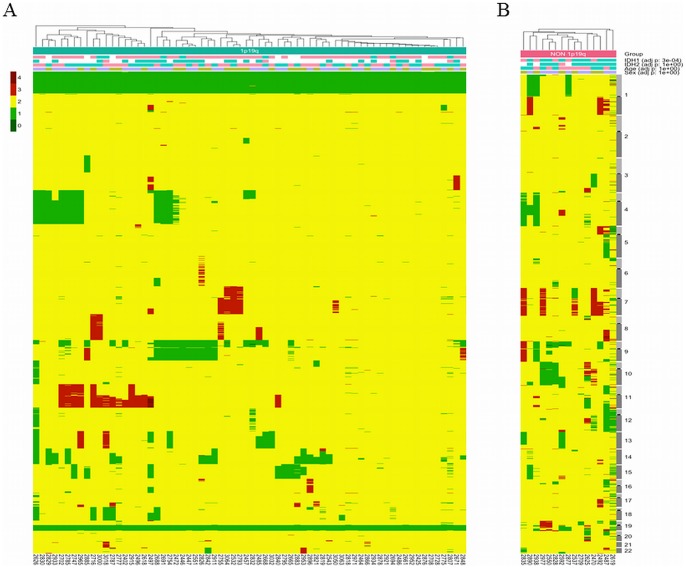
Heat map with genomic profiles of anaplastic oligodendrogliomas. Each column indicates a tumor. Each row indicates a genomic locus. Tumors were clustered based on the Euclidean distance between their copy number vectors. The color code on the left-upper corner indicates the genomic status: yellow, green and red indicate a normal status, loss and gain, respectively. In addition, the *IDH1* mutation (pink indicates mutated *IDH1*/*2*, while *IDH1*/*2* indicates non-mutated *IDH1*/*IDH2*), patient age (blue and pink indicate younger and older, respectively, than the median age of the entire population, 49.9 years old) and patient gender (purple indicates male, while brown indicates female) are reported at the top of the figure. The p-value on the right indicates the distribution of the variables between the 1p19q- and non-1p19q-co-deleted tumors. Panel A. 1p/19q-co-deleted anaplastic oligodendrogliomas, with chromosomes 1 and 19 centromeric breakpoints. Panel B. Non-1p/19q co-deleted anaplastic oligodendrogliomas. The legend is the same as the one used in Panel A.

In the entire cohort, 68/83 (82%) of the tumors contained the 1p/19q-co-deletion with the chromosome 1 and 19 centromeric breakpoints used as a surrogate marker of t(1;19)(q10;p10) (Figure 1, Panel A), while 15/83 (18%) of the samples exhibited other genomic patterns (Figure 1, Panel B). Interestingly, in the latter group, three tumors harbored a whole-chromosome-arm 1p loss without the concurrent whole-chromosome-arm 19q loss, a chromosome 1p centromeric breakpoint or a chromosome 19q centromeric breakpoint. In the same group, an additional three tumors exhibited a whole-chromosome-arm 19q loss without the combined whole-chromosome-arm 1p loss.

**Figure 2:**
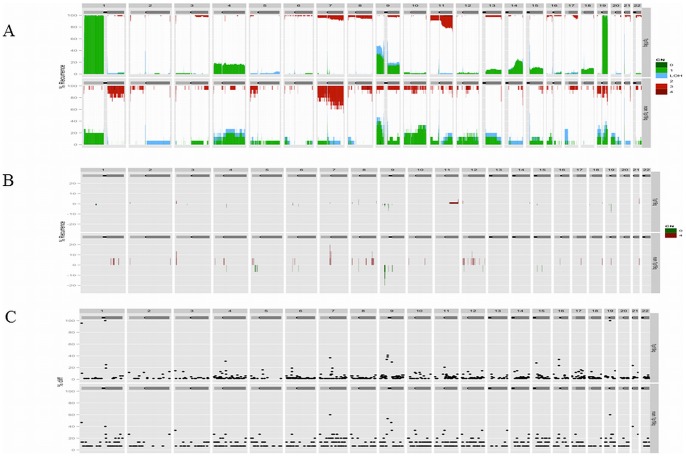
Frequency of genomic alterations in the 1p/19q-co-deleted anaplastic oligodendrogliomas on the top part of the panel and non-1p/19q-co-deleted anaplastic oligodendrogliomas on the bottom part. Panel A. Genomic gain, genomic loss and uniparental disomy are indicated in red, green and blue, respectively. Panel B. High-level amplification and homozygous deletion are indicated in red and green, respectively. Panel C. Genomic breakpoints are indicated with a black dot across the genome.

In the 1p/19q-co-deleted tumors, the most frequent gain was an 11q gain in 19.1% of the cases. The most frequent losses were the deletion of whole chromosome arm 9p, 4p, 4q, 9q and 15q in 32.4%, 17.6%, 16.2%, 14.7%, and 10.3% of the cases, respectively (Figure 2, Panel A, Top part). Sixty-one samples were assessable for *IDH1*, and 53 of them (86.8%) were *IDH1* mutated. The *IDH2* mutation was found in 4/8 of the *IDH1*-intact tumors. Overall, 57/61 (93.4%) of the tumors were *IDH1* or *IDH2* mutated.

In the non-1p/19q-co-deleted group, the most frequent chromosome arm imbalances were a 9p loss, a 7q gain, a 1p loss, a 10p loss or a 10q loss in 40.0%, 26.7%, 20.0%, 20.0% and 20.0% of the samples, respectively (Figure 2, Panel A, Bottom part). All of the samples were assessable for *IDH1*, and 5/15 (33.3%) were *IDH1* mutated. The *IDH2* mutation was found in 1/10 of the *IDH1*-intact tumors. Overall, 6/15 (40.0%) of the tumors were *IDH1* or *IDH2* mutated. The *IDH1* or *IDH2* mutations were more frequently observed in the 1p/19q-co-deleted tumors when compared to the non-1p/19q-co-deleted tumors (p<0.0001) and were mutually exclusive.

### Candidate genomic abnormalities: homozygous deletion and gene amplification

In the 1p/19q-co-deleted samples, a limited number of recurring homozygous deletions were detected (Figure 2-Panel B-Top part and Table 1). Eight loci were found to be homozygously deleted in at least two patients. Interestingly, in one case, a homozygous deletion of *FUBP1* was observed. Additionally, *CDKN2A* was homozygously deleted in one case in the SNP-array analysis and was validated by qPCR. Similarly, recurring gene amplification was rare in the non-1p/19q-co-deleted tumors; eight loci were found to be amplified in at least two patients (Figure 2-Panel B-Bottom part and Table 1). *EGFR* amplification was detected in one case.

**Table 1:** Genomic alterations containing candidate genes in 1p/19q co-deleted anaplastic oligodendrogliomas in at least two tumors (as identified by genoCN).

	Chromosome region	N	Genes
Homozygous deletion	chr19_32455280_32670285	6	
	chr19_32679064_32877033	5	*ZNF507*
	chr9_44683090_44770712	5	
	chr4_69097539_69135491	3	*TMPRSS11B*
	chr9_44779627_45338079	3	*FAM27C*
	chr4_69139402_69258302	2	*YTHDC1*
	chr6_67075448_67105019	2	
	chr9_21963422_22123716	2	*CDKN2B-AS1-CDKN2A-CDKN2B-C9orf53*
High-level amplification	chr21_46815526_46935542	4	*NCRNA00175-SLC19A1-COL18A1*
	chr11_133914145_134445626	3	*VPS26B-GLB1L3-NCAPD3-ACAD8-B3GAT1-LOC283177-THYN1-JAM3-GLB1L2*
	chr21_46746267_46812570	3	
	chr8_39350791_39457081	3	*ADAM3A-ADAM18*
	chr11_133844842_133909403	2	*LOC100128239*
	chr12_21054_213172	2	*LOC100288778-IQSEC3-FAM138D*
	chr3_38411_267992	2	*CHL1*
	chr8_146163558_146264218	2	*ZNF252-TMED10P1-C8orf77-ZNF16*

In the non-1p/19q-co-deleted tumors, the most frequently targeted homozygous deletion was the *CDKN2A* locus (Figure 2, Panel B-Bottom part and Table 2). The *CDKN2A* homozygous deletion was validated in both cases using qPCR. *EGFR* was the most frequently amplified gene; it was detected in three AOD cases using SNP-array analysis and was validated in two of these cases using qPCR (Figure 2-Panel B-Bottom part and Table 2).

**Table 2:** Genomic alterations containing candidate genes in non 1p/19q co-deleted anaplastic oligodendrogliomas in at least two tumors (as identified by genoCN).

	Chromosome region	N	Genes
Homozygous deletion	chr9_22534004_22615342	3	
	chr9_21413394_21951866	2	*IFNE-IFNA1-MIR31-MTAP-LOC554202*
	chr9_21998026_22531137	2	*CDKN2B-AS1-CDKN2B-DMRTA1*
	chr9_22617742_23432605	2	
High-level amplification	chr7_54622953_55307516	3	*EGFR-VSTM2A-SEC61G*
	chr12_56366092_56463559	2	*CDK2-RAB5B-RPS26-IKZF4-SUOX*
	chr3_38411_267992	2	*CHL1*
	chr7_54577787_54620005	2	*VSTM2A*
	chr7_55312776_55466552	2	*LANCL2*
	chr7_61504406_62203847	2	
	chr8_121235610_121468055	2	*MTBP-MRPL13-COL14A1*

### Copy neutral loss of heterozygosity (CNLOH) and CDKN2A expression

**Figure 3:**
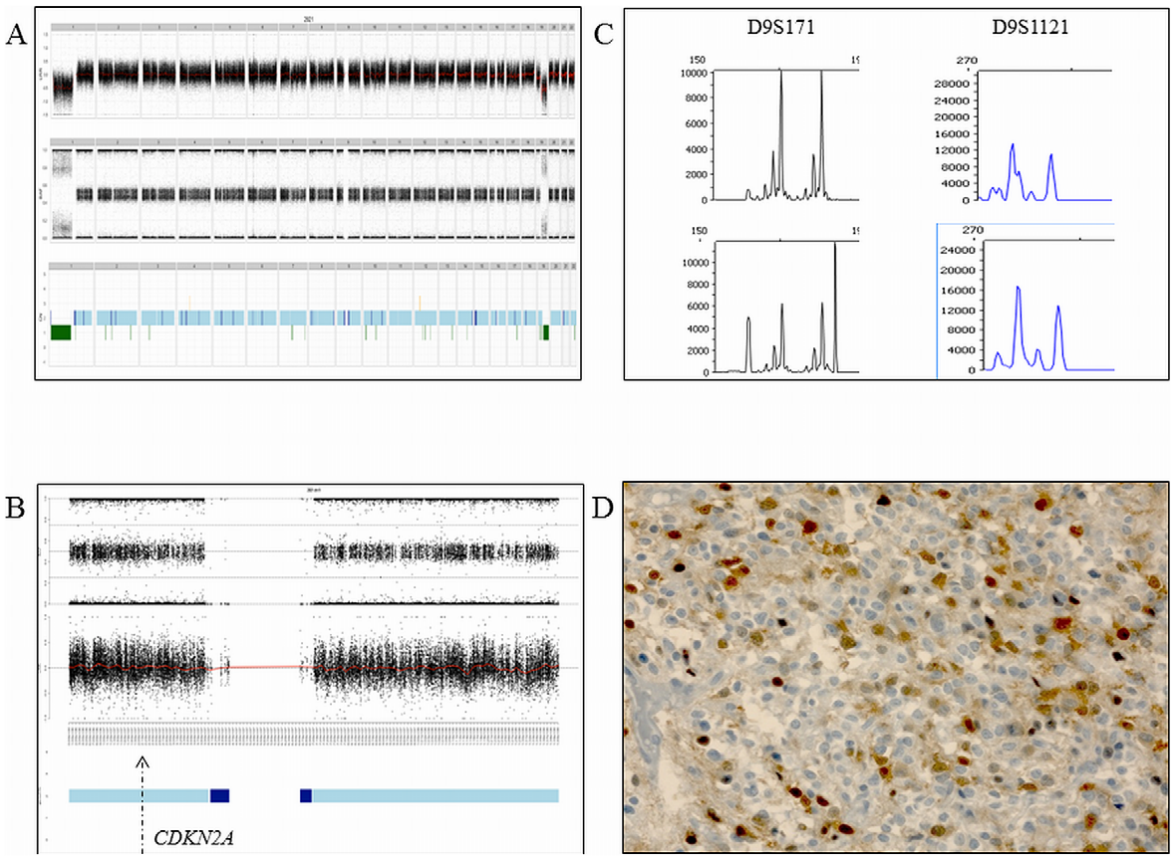
An anaplastic oligodendroglioma with CDKN2A expression and normal *CDKN2A* gene copy number and allelic statuses. Panel A. Top part: Genomic profile with the copy number status. Middle part: Genomic profile with the allelic frequencies. Bottom part: The genomic profile including genomic loss (in green), normal copy number status (light blue) and copy neutral loss of heterozygosity (dark blue). Panel B. Chromosome 9 and the allelic frequencies (the arrow indicates the *CDKNA* locus). Panel C. Microsatellite analysis showing the allelic status of three markers (D9S171 and D9S1121) in the blood DNA (top part) and paired tumor DNA (bottom part) Panel D. CDKN2A expression using immunochemistry.

**Figure 4:**
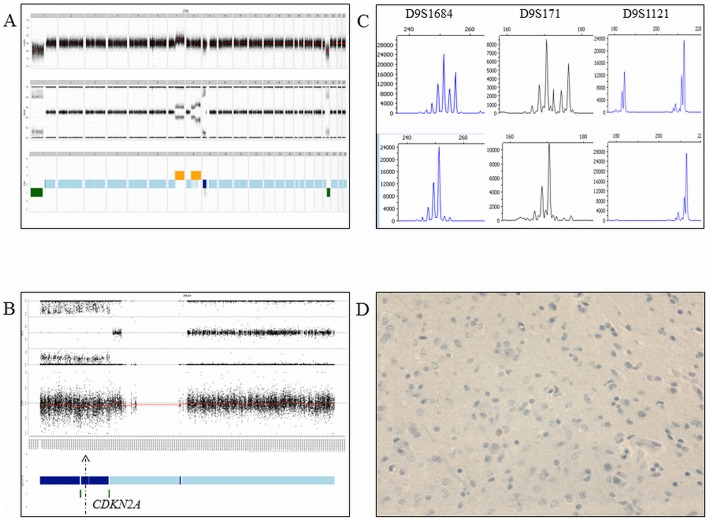
An anaplastic oligodendroglioma with CDKN2A silencing, normal *CDKN2A* gene copy number status and copy neutral loss of heterozygosity. Panel A. Top part: Genomic profile with the copy number status. Middle part: Genomic profile with the allelic frequencies. Bottom part: The genomic profile including genomic loss (in green), normal copy number status (light blue) and copy neutral loss of heterozygosity (dark blue). Panel B. Chromosome 9 and the allelic frequencies (the arrow indicates the *CDKNA* locus). Panel C. Microsatellite analysis showing the allelic status of three markers (D9S1684, D9S171, D9S1121) in the blood DNA (top part) and paired tumor DNA (bottom part). Acquired allelic loss is observed in the tumor DNA Panel D. CDKN2A silenced using immunochemistry.

In the 1p/19q-co-deleted tumors, chromosome arm 9p was the most frequent large-CNLOH-affected chromosome, in 13.2% of cases (Figure 2, Panel A, Top Part). A *CDKN2A* loss of heterozygosity with a normal copy number status was observed in 13.2% of the cases. Correlation of the SNP-array profiling and the CDKN2A expression indicates that the CNLOH is associated with CDKN2A protein silencing in 3/9 of the cases in the 1p/19q-co-deleted AOD tumors (Figures 3 and 4).

In contrast, in the non-1p/19q-co-deleted tumors, CNLOH affected primarily chromosome arm 17p in 20.0% of the cases (Figure 2, Panel A, Bottom part). A *TP53* (located on chromosome arm 17p) loss of heterozygosity with normal copy number status was observed in 26.7% of the cases.

### The co-occurrence of genomic breakpoints

By definition, in the 1p/19q-co-deleted tumors, the most frequent genomic breakpoints were located in chromosomes 1 and 19 in 100% of the tumors (Figure 2, Panel C, Top part and Table 1). Surprisingly, in the non-1p/19q-co-deleted tumors, one of the most common genomic breakpoints also occurred on chromosome 1, close to the centromere (Figure 2, Panel C, Bottom part and Table 2). Interestingly, other recurring breakpoints have been observed in the 1p/19q- and non-1p/19q-co-deleted AOD tumors that disrupt putative candidate genes in AOD oncogenesis (Tables S1 and S2, respectively)

To pinpoint new putative genomic rearrangements, the co-occurrence of the genomic breakpoints was analyzed in both groups. In the 1p/19q-co-deleted tumors, by definition, the chromosome 1 and 19 centromeric breakpoints were observed simultaneously within all of the tumors (Figure S1, Panel A). In the non-1p/19q-co-deleted tumors, the most common co-occurrence of genomic breakpoints involved chromosome 9 (44683090) and chromosome 19 (32455280) in 6/15 cases (Figure S1, Panel B).

### Clinico-molecular correlations

No statistically significant difference was observed between the cohort of patients with 1p/19q-co-deleted tumors *versus* the cohort of patients with non-1p/19q-co-deleted tumors in terms of: (i) sex ratio (1.3 *versus* 1.5, p = 0.9), (ii) median age at diagnosis (50.0 *versus* 49.9 years, p = 0.9) and (iii) median KPS at diagnosis (90 *versus* 90%, p = 0.9).

Although it does not reach statistical significance, patients with 1p/19q co-deleted AOD have longer progression free survival compared to patients with non-1p/19q-co-deleted AOD (median PFS, 846 *versus* 638 days respectively, p = 0.12, Figure S2-Panel A). Patients with 1p/19q co-deleted tumors survive longer than their non-1p/19q co-deleted counterparts (median OS, 886 *versus* 696 days respectively, p = 0.04, Figure S2-Panel B)

## Discussion

AOD forms a heterogeneous subgroup of diffuse gliomas with both a heterogeneous biology and clinical course. In addition to the clinical, pathological and radiological studies, pivotal biological studies have identified critical molecular abnormalities with both clinical and biological relevance in AOD [4], [5], [7], [14], [15]. The 1p/19q-co-deletion has been shown to be recurring and is associated with better outcomes in AOD patients [4]–[7]. Recently, the *IDH1*/*IDH2*, *CIC* and *FUBP1* mutations have been discovered in 100%, ∼80% and ∼25% of the 1p/19q-co-deleted AOD tumors [13]–[15], [23]. At the epigenomic level, the hypermethylated phenotype has been shown to be associated with *IDH* mutation and better outcomes in AOD patients [24]–[27]. Therefore, molecular characterization contributes not only to the basic dissection of the AOD group but also to the identification of new relevant biomarkers.

However, to our knowledge, the present study is the first to investigate a large prospective cohort of centrally validated AOD cases, using a high-resolution SNP array. One of the advantages of SNP arrays is their ability to assess not only genomic copy number variations but also copy neutral loss of heterozygosity (uniparental disomy).

Using this strategy, we have identified novel focal- and large-genomic abnormalities in AOD. Frequent CNLOH targeting of chromosome arm 9p and the *CDKN2A* locus have been observed in the 1p/19q-co-deleted tumors. Therefore, the present study shows that chromosome arm 9p and the *CDKN2A* locus are by far the most frequently altered genomic regions in the 1p/19q-co-deleted tumors, either through genomic loss (32.4%) or CNLOH (13.2%). In patients with *CDKN2A* CNLOH, we found that the CDKN2A expression was silenced in 3/9 patients, suggesting that CNLOH is one mechanism of *CDKN2A* silencing and expression regulation. In cancer cells, CNLOH has been shown to regulate gene expression according to the parental gene duplicate (regardless of imprinting) [28]. Interestingly, a homozygous deletion of *FUBP1* was detected in one case. These data combined with the recent work of Bettogowda et al. suggest that *FUBP1* has a putative tumor suppressor role in oligodendrogliomagenesis. In addition, the high resolution genome-wide analysis conducted in the present study highlighted multiple novel focal genomic abnormalities containing putative genes involved in AOD oncogenesis. Further investigations are required to specify these candidate genes and their role in the biology of AOD.

Our study confirms that despite a rigorously controlled homogeneous pathological aspect, AOD is a heterogeneous subgroup of tumors in terms of its molecular features. The majority of tumors exhibited the 1p/19q-co-deletion (82%), while a minority of cases (18%) harbored molecular alterations frequently observed in high-grade astrocytic tumors (i.e., *EGFR* amplification, chromosome 10 loss). The molecular status has been validated in a prospective clinical trial as a critical prognosis indicator in AOD patients [4], [5], supporting the implementation of molecular testing, particularly the 1p/19q status, combined with pathological features in AOD diagnosis. The best technique for the detection of the 1p/19q-co-deletion is still debated. Our study supports whole chromosome screening of chromosomes 1 and 19 in order to reliably detect the 1p/19q-co-deletion, with the centromeric breakpoints as a surrogate marker of t(1;19)(q10;p10), since limited or isolated 1p and 19q losses have also been observed in “false” 1p/19q-co-deleted tumors [8], [9], [29], [30].

Because t(1;19)(q10;p10) is a genomic hallmark of oligodendrogliomas and the putative fusion gene has not yet been identified [31], a part of the present work was focused on the genomic breakpoints and their occurrence in order to pinpoint putative chimeric genes. Multiple genes were found to be disrupted by chromosome breakpoints, though additional molecular studies are required to provide a more in-depth investigation of the “disrupted” genes and the potential fusion gene resulting from these genomic breakpoints co-occurrences.

The *IDH1*/*2* mutations, as previously shown, were strongly associated with the 1p/19q-co-deletion (93.4% of the 1p/19q-co-deleted AOD cases exhibited the *IDH1*/*2* mutation). We previously reported that all of the 1p/19q-co-deleted tumors are *IDH1/2* mutated [23]. This minor discrepancy might be related to tumor heterogeneity.

Finally, the limited number of non 1p/19q-co-deleted tumors and the short follow-up do not allow robust prognostic analysis so far. Nonetheless, as expected, patients with 1p/19q co-deleted tumors survive longer than patients whom tumor does not harbor this biomarker.

In conclusion, high resolution SNP array analysis was used in a prospective centrally reviewed series of AOD-identified novel copy number abnormalities containing putative candidate genes and identified CNLOH as a novel recurrent genomic abnormality in AOD. In addition to neuropathological examination, integration of the copy number abnormality data with other OMICS data will aid in specifying the genetic portraits of the different entities encompassed in the AOD group, aiding in a more accurate histomolecular diagnosis and a better understanding of AOD oncogenesis.

## Supporting Information

Figure S1 The co-occurrence of genomic breakpoints in the 1p/19q-co-deleted anaplastic oligodendrogliomas (Panel A) and in the non-1p/19q-co-deleted anaplastic oligodendrogliomas (Panel B). Blue and green indicate the absence and presence of chromosome breakpoints, respectively. The tumor sample and broken genomic regions are reported at the bottom and the right of the figure, respectively. The left dendrogram indicates a co-occurring breakpoint and the top dendrogram indicates tumors with similar genomic breakpoint patterns.
(TIF)

Figure S2 Kaplan-Meier curves comparing progression free survival (PFS, Panel A) and overall survival (OS, Panel B) of patients with 1p/19q co-deleted anaplastic oligodendroglioma (continuous line) *versus* patients with non-1p/19q-co-deleted tumors (broken line). Although a trend is observed, no statistically significant difference is observed for PFS. OS between both group is statistically different (p = 0.04).
(TIF)

Table S1 Genomic breakpoints in 1p/19q codeleted anaplastic oligodendroglioma.
(DOC)

Table S2 Genomic breakpoints in non 1p/19q codeleted anaplastic oligodendroglioma.
(DOC)
