# Correction: SNP Array Analysis Reveals Novel Genomic Abnormalities Including Copy Neutral Loss of Heterozygosity in Anaplastic Oligodendrogliomas

**DOI:** 10.1371/annotation/27807b78-9c79-414a-a47e-fb3eca621be4

**Published:** 2013-05-08

**Authors:** Ahmed Idbaih, François Ducray, Caroline Dehais, Célia Courdy, Catherine Carpentier, Simon de Bernard, Emmanuelle Uro-Coste, Karima Mokhtari, Anne Jouvet, Jérôme Honnorat, Olivier Chinot, Carole Ramirez, Patrick Beauchesne, Alexandra Benouaich-Amiel, Joël Godard, Sandrine Eimer, Fabrice Parker, Emmanuelle Lechapt-Zalcman, Philippe Colin, Delphine Loussouarn, Thierry Faillot, Phong Dam-Hieu, Selma Elouadhani-Hamdi, Luc Bauchet, Olivier Langlois, Caroline Le Guerinel, Denys Fontaine, Elodie Vauleon, Philippe Menei, Marie Janette Motsuo Fotso, Christine Desenclos, Pierre Verrelle, François Ghiringhelli, Georges Noel, François Labrousse, Antoine Carpentier, Frédéric Dhermain, Jean-Yves Delattre, Dominique Figarella-Branger, POLA Network

There was an error in the name of the 32nd author.

The correct name is: Pierre Verrelle 

