# Supplementary figures and images for: SNP Array Analysis Reveals Novel Genomic Abnormalities Including Copy Neutral Loss of Heterozygosity in Anaplastic Oligodendrogliomas
Source: PLoS One. 2012 Oct 10;7(10):e45950. doi: 10.1371/journal.pone.0045950 (PMC3468603; doi:10.1371/journal.pone.0045950)

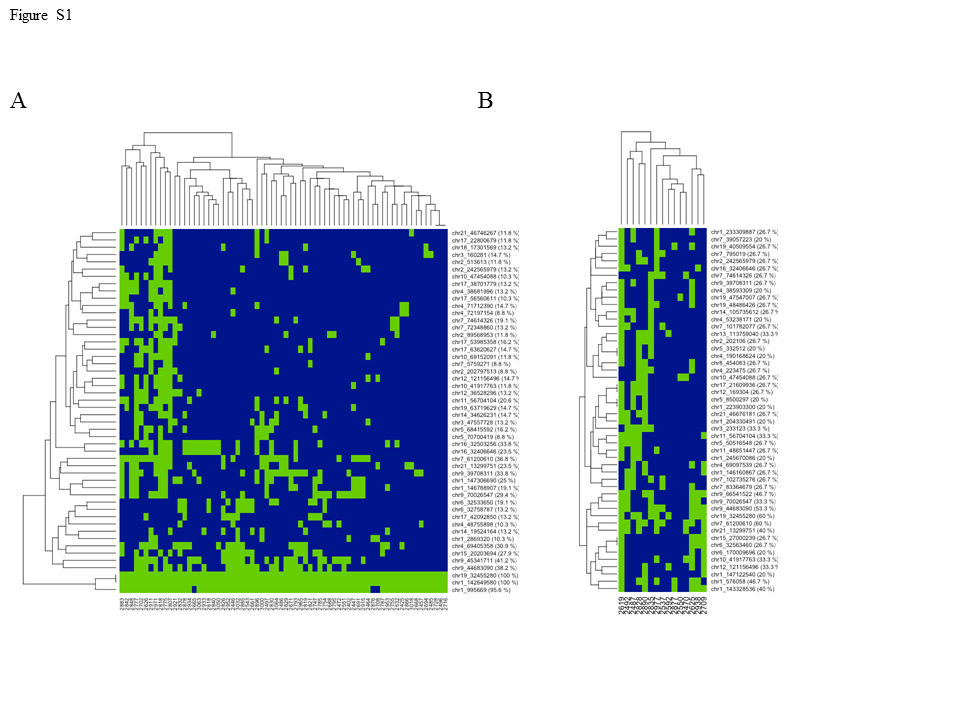

Supplement: Figure S1 — The co-occurrence of genomic breakpoints in the 1p/19q-co-deleted anaplastic oligodendrogliomas (Panel A) and in the non-1p/19q-co-deleted anaplastic oligodendrogliomas (Panel B). Blue and green indicate the absence and presence of chromosome breakpoints, respectively. The tumor sample and broken genomic regions are reported at the bottom and the right of the figure, respectively. The left dendrogram indicates a co-occurring breakpoint and the top dendrogram indicates tumors with similar genomic breakpoint patterns. (TIF) [file pone.0045950.s001.tif]

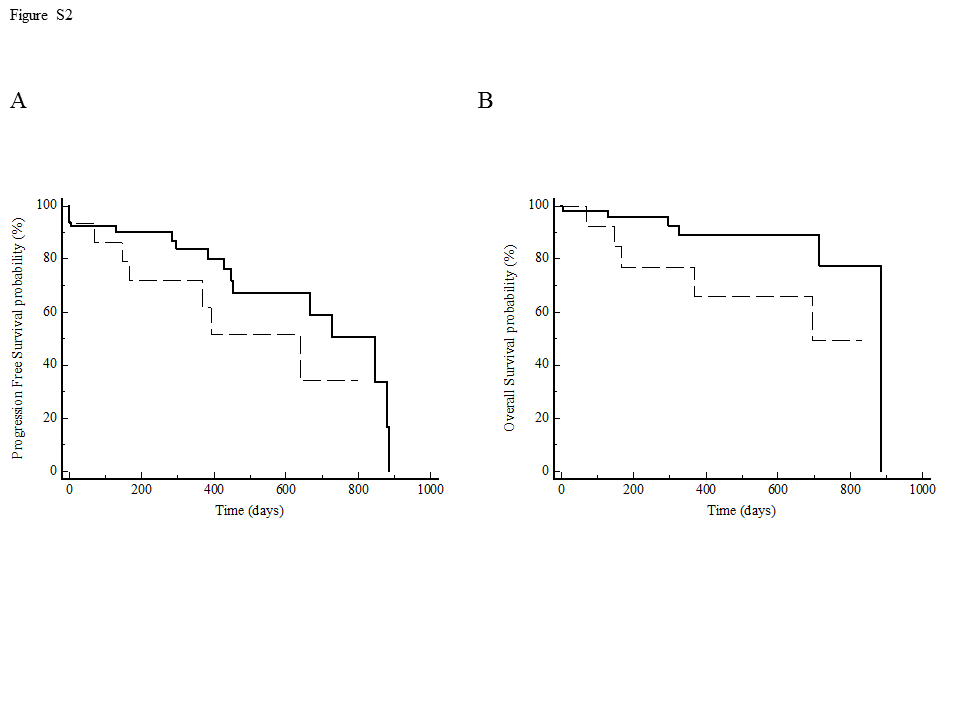

Supplement: Figure S2 — Kaplan-Meier curves comparing progression free survival (PFS, Panel A) and overall survival (OS, Panel B) of patients with 1p/19q co-deleted anaplastic oligodendroglioma (continuous line) versus patients with non-1p/19q-co-deleted tumors (broken line). Although a trend is observed, no statistically significant difference is observed for PFS. OS between both group is statistically different (p = 0.04). (TIF) [file pone.0045950.s002.tif]
