# Supplementary material for: SNP Array Analysis Reveals Novel Genomic Abnormalities Including Copy Neutral Loss of Heterozygosity in Anaplastic Oligodendrogliomas
Source: PLoS One. 2012 Oct 10;7(10):e45950. doi: 10.1371/journal.pone.0045950 (PMC3468603; doi:10.1371/journal.pone.0045950)
Supplement: Table S1 — Genomic breakpoints in 1p/19q codeleted anaplastic oligodendroglioma. (DOC) [file pone.0045950.s003.doc]

Table S1. Genomic breakpoints in 1p/19q codeleted anaplastic oligodendroglioma.

| Chromosome region | N (%) | Genes |
| --- | --- | --- |
| chr1_142649580 | 100 | LOC100130000 - LOC100286793 - PPIAL4A - FLJ39739 - NBPF9 - PPIAL4G - SRGAP2P2 - PPIAL4B - PPIAL4C - FAM72D - LOC728855 - LOC728875 - C1orf152 |
| chr19_32455280 | 100 | CEBPA - CEBPG - SLC7A9 - WDR88 - DPY19L3 - ZNF507 - RGS9BP - NUDT19 - LRP3 - PEPD - GPATCH1 - SLC7A10 - TSHZ3 - CHST8 - KCTD15 - LOC80054 - ANKRD27 - CCDC123 - RHPN2 - C19orf2 - PDCD5 - C19orf40 - TDRD12 - DKFZp566F0947 - ZNF536 |
| chr1_995669 | 95.6 | LOC100128003 - LOC100129534 - FLJ39609 - LOC100132062 - LOC100132287 - LOC100133331 - LOC100288069 - RER1 - LOC115110 - ACAP3 - UBE2J2 - PUSL1 - B3GALT6 - C1orf93 - ACTRT2 - MIB2 - SAMD11 - LOC148413 - CALML6 - DVL1 - C1orf86 - ATAD3C - TTLL10 - GABRD - NOC2L - OR4F3 - GNB1 - FAM41C - SSU72 - KLHL17 - C1orf70 - TMEM52 - AGRN - FAM132A - HES5 - MIR200A - MIR200B - FLJ42875 - LOC441869 - SDF4 - PEX10 - MXRA8 - CPSF3L - C1orf159 - AURKAIP1 - MRPL20 - ATAD3A - PANK4 - MIR429 - PRKCZ - HES4 - SCNN1D - PRDM16 - FAM138F - LOC643837 - TMEM88B - FAM138A - VWA1 - SKI - NADK - WASH7P - FAM138C - CDK11A - SLC35E2B - TNFRSF4 - OR4F29 - MMEL1 - OR4F5 - NCRNA00115 - MORN1 - GLTPD1 - OR4F16 - CCNL2 - TAS1R3 - ATAD3B - PLEKHN1 - C1orf170 - MMP23B - MMP23A - KIAA1751 - TNFRSF14 - TNFRSF18 - ISG15 - PLCH2 - CDK11B - SLC35E2 |
| chr9_45341711 | 41.2 | FAM27C - KGFLP1 - FAM75A6 - FAM27A |
| chr9_44683090 | 38.2 | FOXD4L2 - FAM27C - FAM95B1 - FOXD4L4 - FAM75A6 - ANKRD20A3 - ANKRD20A2 - AQP7P3 - FAM27A - LOC642929 |
| chr7_61200610 | 36.7 | LOC100287704 - LOC100287834 - MIR4283-2 - MIR4283-1 - LOC643955 |
| chr9_39708311 | 33.8 | FAM201A - RG9MTD3 - ALDH1B1 - FRMPD1 - ANKRD18A - ZNF658 - FAM75A7 - IGFBPL1 - FAM74A1 - ZNF658B - EXOSC3 - FAM75A2 - SHB - FAM75A1 - LOC653501 - FAM75A3 - FAM75A5 - FAM74A3 - DCAF10 - CNTNAP3 - MCART1 |
| chr16_32503256 | 33.8 | KIAA0664L3 - MIR762 - BCKDK - ZNF267 - SRCAP - STX1B - ZNF689 - ZNF720 - COX6A2 - ZNF785 - ZNF688 - PRSS36 - CTF1 - ZNF629 - TP53TG3 - FUS - PYDC1 - NCRNA00095 - ZNF843 - PYCARD - PRSS53 - ITGAD - ITGAL - ITGAM - ITGAX - SLC6A10P - LOC390705 - CSDAP1 - HERC2P4 - TRIM72 - AHSP - PHKG2 - FBXL19 - PRSS8 - UBE2MP1 - FBRS - C16orf58 - SLC5A2 - ZNF747 - SNORA30 - STX4 - TGFB1I1 - TP53TG3B - PRR14 - VKORC1 - ZNF768 - ZNF668 - ARMC5 - HSD3B7 - MYST1 - C16orf93 - ZNF764 - BCL7C - ORAI3 - ZNF646 - SETD1A - RNF40 |
| chr4_69405358 | 30.8 | FTLP10 - CSN1S2BP - CENPC1 - UGT2B11 - SMR3B - UGT2A1 - TMPRSS11B - CSN1S1 - CSN2 - CSN3 - C4orf7 - STAP1 - SMR3A - SULT1B1 - GNRHR - CSN1S2AP - TMPRSS11E - HTN1 - HTN3 - TMPRSS11A - TMPRSS11F - SYT14L - TMPRSS11BNL - C4orf40 - AMTN - MUC7 - UGT2B28 - ODAM - LOC550112 - UBA6 - UGT2A2 - PROL1 - LOC644759 - STATH - SULT1E1 - UGT2B4 - UGT2B7 - UGT2B10 - UGT2B15 - UGT2B17 - UGT2A3 - CABS1 - YTHDC1 - TMPRSS11D |
| chr9_70026547 | 29.4 | FOXD4L2 - LOC100132352 - LOC100133920 - FAM122A - C9orf71 - CBWD5 - FXN - FOXD4L3 - FOXD4L4 - LOC440896 - CBWD3 - PGM5 - PRKACG - LOC572558 - PGM5P2 - LOC642236 - CBWD6 - FOXD4L6 - FOXD4L5 - ANKRD20A4 - PIP5K1B - FAM189A2 - TJP2 |
| chr15_20203694 | 27.9 | HERC2P3 - POTEB - NF1P1 - BCL8 - LOC646214 - CXADRP2 - GOLGA6L6 - GOLGA8C" |
| chr1_147306690 | 25 | NBPF10 - PIAS3 - POLR3C - TXNIP - CD160 - GNRHR2 - LIX1L - HFE2 - ANKRD35 - PPIAL4A - PDIA3P - NBPF11 - NUDT17 - FMO5 - NBPF14 - GJA5 - GJA8 - RNF115 - NBPF15 - ANKRD34A - FLJ39739 - LOC388692 - ACP6 - GPR89B - PDZK1 - PRKAB2 - BCL9 - PPIAL4D - LOC645166 - PPIAL4B - GPR89A - NBPF24 - GPR89C - NBPF16 - PDZK1P1 - PPIAL4F - LOC728989 - PPIAL4E - POLR3GL - ITGA10 - PEX11B - CHD1L - RBM8A |
