# Supplementary material for: SNP Array Analysis Reveals Novel Genomic Abnormalities Including Copy Neutral Loss of Heterozygosity in Anaplastic Oligodendrogliomas
Source: PLoS One. 2012 Oct 10;7(10):e45950. doi: 10.1371/journal.pone.0045950 (PMC3468603; doi:10.1371/journal.pone.0045950)
Supplement: Table S2 — Genomic breakpoints in non 1p/19q codeleted anaplastic oligodendroglioma. (DOC) [file pone.0045950.s004.doc]

Table S2. Genomic breakpoints in non 1p/19q codeleted anaplastic oligodendroglioma

| Chromosome region | N (%) | Genes |
| --- | --- | --- |
| chr7_61200610 | 60 | "LOC100287704 - LOC100287834 - MIR4283-2 - MIR4283-1 - LOC643955" |
| chr19_32455280 | 60 | "CEBPA - CEBPG - SLC7A9 - WDR88 - DPY19L3 - ZNF507 - RGS9BP - NUDT19 - LRP3 - PEPD - GPATCH1 - SLC7A10 - TSHZ3 - CHST8 - KCTD15 - LOC80054 - ANKRD27 - CCDC123 - RHPN2 - C19orf2 - PDCD5 - C19orf40 - TDRD12 - DKFZp566F0947 - ZNF536" |
| chr9_44683090 | 53.3 | "FOXD4L2 - FAM27C - FAM95B1 - FOXD4L4 - FAM75A6 - ANKRD20A3 - ANKRD20A2 - AQP7P3 - FAM27A - LOC642929" |
| chr1_576058 | 46.7 | "LOC100128003 - LOC100129534 - FLJ39609 - LOC100132062 - LOC100132287 - LOC100133331 - LOC100288069 - RER1 - LOC115110 - ACAP3 - UBE2J2 - PUSL1 - B3GALT6 - C1orf93 - MIB2 - SAMD11 - LOC148413 - CALML6 - DVL1 - C1orf86 - ATAD3C - TTLL10 - GABRD - NOC2L - OR4F3 - GNB1 - FAM41C - SSU72 - KLHL17 - C1orf70 - TMEM52 - AGRN - FAM132A - HES5 - MIR200A - MIR200B - LOC441869 - SDF4 - PEX10 - MXRA8 - CPSF3L - C1orf159 - AURKAIP1 - MRPL20 - ATAD3A - PANK4 - MIR429 - PRKCZ - HES4 - SCNN1D - FAM138F - LOC643837 - TMEM88B - FAM138A - VWA1 - SKI - NADK - WASH7P - FAM138C - CDK11A - SLC35E2B - TNFRSF4 - OR4F29 - MMEL1 - OR4F5 - NCRNA00115 - MORN1 - GLTPD1 - OR4F16 - CCNL2 - TAS1R3 - ATAD3B - PLEKHN1 - C1orf170 - MMP23B - MMP23A - KIAA1751 - TNFRSF14 - TNFRSF18 - ISG15 - PLCH2 - CDK11B - SLC35E2" |
| chr9_66541522 | 46.7 | "FAM27B - LOC100133920 - FAM75A7 - AQP7P1 - AQP7P2 - FAM74A4 - ANKRD20A3 - LOC442421 - LOC642236 - FAM74A2 - FAM75A5 - ANKRD20A1" |
| chr1_143328536 | 40 | "LOC100130000 - NBPF10 - LOC100286793 - PPIAL4A - NOTCH2NL - FLJ39739 - NBPF9 - PPIAL4G - SRGAP2P2 - PPIAL4B - PPIAL4C - FAM72D - LOC728855 - LOC728875 - C1orf152 - SEC22B - PDE4DIP" |
| chr21_13299751 | 40 | "MIR3156-3 - ANKRD30BP2 - POTED - C21orf15" |
| chr3_233123 | 33.3 | "CHL1 - CNTN4 - CNTN6" |
| chr9_70026547 | 33.3 | "FOXD4L2 - LOC100132352 - LOC100133920 - FAM122A - C9orf71 - CBWD5 - FXN - FOXD4L3 - FOXD4L4 - LOC440896 - CBWD3 - PGM5 - PRKACG - LOC572558 - PGM5P2 - LOC642236 - CBWD6 - FOXD4L6 - FOXD4L5 - ANKRD20A4 - PIP5K1B - FAM189A2 - TJP2" |
| chr10_41917763 | 33.3 | "ZNF37BP - RASGEF1A - HNRNPF - LOC441666 - FXYD4 - CSGALNACT2 - RET - ZNF33B - LOC84856 - BMS1" |
| chr11_56704104 | 33.3 | "GLYAT - PRG3 - OR5I1 - CLP1 - OR8I2 - CNTF - CTNND1 - APLNR - OR8U1 - OR4C16 - OR4C11 - OR4S2 - OR4C6 - OR5D14 - OR5L1 - OR5D18 - OR7E5P - OR5AS1 - OR8K5 - OR5T2 - OR8H1 - OR8K3 - OR8J1 - OR5R1 - OR5M3 - OR5M8 - OR5M11 - OR5AR1 - OR5AK4P - LRRC55 - SMTNL1 - YPEL4 - MED19 - OR6Q1 - OR9I1 - OR9Q1 - OR9Q2 - OR1S2 - OR1S1 - OR10Q1 - OR5B17 - OR5B21 - GLYATL2 - ZDHHC5 - OR5L2 - TIMM10 - C11orf31 - OR10AG1 - OR5J2 - OR9G4 - LOC283194 - SLC43A3 - OR5F1 - OR5AP2 - RTN4RL2 - ZFP91-CNTF - OR5D13 - OR5D16 - OR5W2 - OR8H2 - OR8H3 - OR5T3 - OR5T1 - OR8K1 - OR5M9 - OR5M10 - OR5M1 - OR9G1 - OR5AK2 - OR5B2 - OR5B12 - MIR130A - OR5B3 - P2RX3 - OR8U8 - OR9G9 - TMX2 - PRG2 - BTBD18 - SSRP1 - SERPING1 - TRIM48 - ZFP91 - OR8J3 - OR4P4 - OR4C15 - OR4A16 - OR4A15 - OR10W1 - SPRYD5 - SLC43A1 - TNKS1BP1 - UBE2L6 - LPXN" |
| chr12_121156496 | 33.3 | "MIR1178 - CAMKK2 - GCN1L1 - RAB35 - CIT - SPPL3 - COX6A1 - TMEM120B - WDR66 - LOC144742 - CCDC60 - MLXIP - SETD1B - SIRT4 - LRRC43 - HSPB8 - MORN3 - NME2P1 - GATC - NCRNA00262 - HPD - LOC338799 - ACADS - IL31 - TMEM233 - MSI1 - P2RX4 - P2RX7 - POP5 - ANAPC5 - TRIAP1 - PLA2G1B - RHOF - ZCCHC8 - PRKAB1 - DIABLO - PSMD9 - PXN - BCL7A - RPLP0 - CLIP1 - C12orf43 - VPS33A - RSRC2 - HNF1A - B3GNT4 - RNF34 - COQ5 - SRRM4 - KDM2B - UNC119B - ORAI1 - OASL - DYNLL1 - SRSF9 - CCDC64 - CABP1 - KNTC1 - MLEC - RNF10" |
| chr13_113759040 | 33.3 | "LOC100130386 - LOC100289410 - TUBGCP3 - ADPRHL1 - C13orf16 - C13orf28 - F7 - F10 - RASA3 - ATP11A - MCF2L - GAS6 - ZNF828 - FAM70B - LAMP1 - C13orf35 - ATP4B - TMCO3 - DCUN1D2 - PCID2 - GRK1 - FLJ44054 - UPF3A - SOX1 - TFDP1 - GRTP1 - CUL4A - PROZ - ARHGEF7 - CDC16" |
| chr1_146160867 | 26.7 | "NBPF10 - PIAS3 - POLR3C - TXNIP - CD160 - GNRHR2 - LIX1L - HFE2 - ANKRD35 - PPIAL4A - PDIA3P - NBPF11 - NUDT17 - FMO5 - NBPF14 - GJA5 - GJA8 - RNF115 - ANKRD34A - NOTCH2NL - FLJ39739 - NBPF9 - ACP6 - GPR89B - PDZK1 - PRKAB2 - BCL9 - PPIAL4B - GPR89A - PPIAL4C - LOC728855 - LOC728875 - NBPF24 - GPR89C - PDZK1P1 - LOC728989 - C1orf152 - POLR3GL - ITGA10 - PEX11B - SEC22B - CHD1L - PDE4DIP - RBM8A" |
| chr1_233309887 | 26.7 | "LOC100287814 - NCRNA00184 - TSNAX-DISC1 - C1orf131 - SLC35F3 - EXOC8 - RBM34 - DISC2 - DISC1 - IRF2BP2 - KCNK1 - C1orf31 - TRIM67 - EGLN1 - KIAA1383 - SIPA1L2 - SNRPD2P2 - SNORA14B - TARBP1 - TSNAX - PCNXL2 - C1orf124 - NTPCR - GNPAT - KIAA1804 - TOMM20" |
| chr2_202106 | 26.7 | "TMEM18 - MYT1L - SH3YL1 - FAM150B - LOC339822 - ACP1 - SNTG2 - FAM110C - TPO - PXDN" |
| chr2_242565979 | 26.7 | "PP14571 - BOK-AS - STK25 - CAPN10 - NEU4 - MTERFD2 - OTOS - MYEOV2 - OR6B3 - DTYMK - AGXT - LOC200772 - PASK - ATG4B - SNED1 - GPC1 - C2orf85 - DUSP28 - GPR35 - HDLBP - AQP12A - OR6B2 - MIR149 - NDUFA10 - SEPT2 - ANO7 - THAP4 - ANKMY1 - PDCD1 - KIF1A - PPP1R7 - RNPEPL1 - GAL3ST2 - PRR21 - AQP12B - BOK - D2HGDH - LOC728323 - C2orf54 - ING5 - FARP2" |
| chr4_223475 | 26.7 | "MIR943 - LOC100129917 - LOC100130872 - TMED11P - MAEA - PCGF3 - SPON2 - TACC3 - CPLX1 - SLC26A1 - CTBP1 - ZNF595 - FAM53A - DGKQ - ZNF721 - FGFR3 - ZNF718 - GAK - CRIPAK - RNF212 - NAT8L - IDUA - POLN - LETM1 - C4orf48 - MYL5 - PDE6B - ATP5I - FGFRL1 - PIGG - KIAA1530 - ZNF876P - ZNF732 - SCARNA22 - WHSC1 - WHSC2 - ZNF141 - SLBP - ABCA11P - MFSD7 - TMEM175 - C4orf42 - TMEM129" |
| chr4_69097539 | 26.7 | "FTLP10 - CSN1S2BP - CENPC1 - UGT2B11 - UGT2A1 - TMPRSS11B - CSN1S1 - CSN2 - C4orf7 - STAP1 - SULT1B1 - GNRHR - CSN1S2AP - TMPRSS11E - HTN1 - HTN3 - TMPRSS11A - TMPRSS11F - SYT14L - TMPRSS11BNL - C4orf40 - UGT2B28 - ODAM - LOC550112 - UBA6 - UGT2A2 - LOC644759 - STATH - SULT1E1 - UGT2B4 - UGT2B7 - UGT2B10 - UGT2B15 - UGT2B17 - UGT2A3 - YTHDC1 - TMPRSS11D" |
| chr5_50516548 | 26.7 | "EMB - LOC257396 - ISL1 - ITGA1 - ITGA2 - MOCS2 - PELO - PARP8" |
| chr6_32563460 | 26.7 | "PSORS1C3 - MIR1275 - MIR1236 - FLOT1 - CDSN - PFDN6 - AGPAT1 - C6orf10 - HCP5 - EHMT2 - NUDT3 - NRM - IP6K3 - CLIC1 - COL11A2 - DPCR1 - ATF6B - CSNK2B - CYP21A2 - DAXX - PSORS1C1 - PSORS1C2 - KIAA1949 - AGER - DOM3Z - AIF1 - TUBB - C6orf1 - LEMD2 - ZBTB9 - ZBTB12 - C6orf136 - DDAH2 - HCG27 - SPDEF - NCR3 - LY6G6F - SNORD52 - SNORD48 - HCG22 - RNF5P1 - MRPS18B - C6orf15 - GRM4 - GTF2H4 - PACSIN1 - HLA-B - HLA-C - HLA-DMA - HLA-DMB - HLA-DOA - HLA-DOB - HLA-DPA1 - HLA-DPB1 - HLA-DPB2 - HLA-DQA1 - HLA-DQA2 - HLA-DQB1 - HLA-DQB2 - HLA-DRA - HLA-DRB1 - HLA-DRB5 - HLA-DRB6 - HMGA1 - HSPA1A - HSPA1B - HSPA1L - HCG26 - ITPR3 - KIFC1 - SFTA2 - MUC21 - MCCD1 - C6orf26 - C6orf227 - LTA - LTB - MIR219-1 - MICB - MLN - MSH5 - GGNBP1 - NEU1 - NFKBIL1 - NOTCH4 - C6orf48 - PBX2 - CUTA - PHF1 - ATP6V1G2 - CCHCR1 - POU5F1 - PPP1R10 - APOM - BTNL2 - PSMB8 - PSMB9 - VARS2 - BAK1 - LSM2 - C6orf47 - LY6G5B - LY6G6D - RGL2 - RING1 - BRD2 - RNF5 - RPS10 - RPS18 - RXRB - CFB - VPS52 - GPSM3 - FKBPL - C6orf106 - SKIV2L - LYPLA2P1 - SNORA38 - TAP1 - TAP2 - TAPBP - SNORD84 - SNORD117 - TCF19 - TNF - TNXA - TNXB - C2 - C4A - C4B - VARS - DDR1 - LY6G6E - PRRC2A - BAG6 - GPANK1 - DDX39B - ABHD16A - SLC39A7 - HSD17B8 - RDBP - LST1 - ATAT1 - SLC44A4 - C6orf27 - C6orf25 - LY6G6C - LY6G5C - PRRT1 - EGFL8 - C6orf125 - DHX16 - B3GALT4 - SYNGAP1 - STK19 - IER3 - WDR46 - ZBTB22 - PPT2 - MDC1" |
| chr7_795019 | 26.7 | "LOC100288524 - ADAP1 - KIAA1908 - GPR146 - AMZ1 - TMEM184A - BAAT1 - IQCE - SUN1 - TFAMP1 - INTS1 - GNA12 - GPER - SNX8 - FTSJ2 - UNCX - ELFN1 - LFNG - FLJ44511 - LOC442497 - MIR339 - NUDT1 - PDGFA - GET4 - CYP2W1 - HEATR2 - CHST12 - PRKAR1B - FAM20C - MAFK - MICALL2 - TTYH3 - MAD1L1 - PSMG3 - C7orf50 - EIF3B - ZFAND2A - COX19" |
| chr7_74614326 | 26.7 | "LOC100093631 - POM121C - LOC100133091 - MIR4284 - CCL26 - DTX2 - WBSCR22 - WBSCR28 - TRIM50 - CLDN4 - CLDN3 - SRCRB4D - WBSCR27 - VPS37D - NSUN5P1 - WBSCR26 - ELN - SRRM3 - POMZP3 - TBL2 - GTF2I - GTF2IP1 - HIP1 - HSPB1 - TRIM73 - TRIM74 - SPDYE8P - GATSL1 - GTF2IRD2B - LIMK1 - GTF2IRD2P1 - MDH2 - PMS2P11 - STAG3L2 - SPDYE5 - MLXIPL - STYXL1 - PMS2L2 - PMS2P5 - PMS2P3 - STAG3L1 - POR - NSUN5 - RHBDD2 - RFC2 - FDPSL2A - CCL24 - NCF1 - NCF1B - NCF1C - SNORA14A - STX1A - MIR590 - GATSL2 - EIF4H - CLIP2 - LAT2 - YWHAG - ZP3 - UPK3B - WBSCR16 - FZD9 - ABHD11 - TMEM120A - GTF2IRD2 - DNAJC30 - FKBP6 - BAZ1B - BCL7B - GTF2IRD1" |
| chr7_83364679 | 26.7 | "SEMA3A - SEMA3D - PCLO - HGF - CACNA2D1 - SEMA3E" |
| chr7_101782077 | 26.7 | "RPL19P12 - UPK3BL - SPDYE2L - SAP25 - MIR4285 - MUC12 - RASA4 - LRRC17 - POP7 - ZNHIT1 - SH2B2 - STAG3 - AP1S1 - EMID2 - MUC17 - CUX1 - EPHB4 - EPO - C7orf47 - LRWD1 - FAM185A - FBXL13 - NAPEPLD - C7orf51 - CLDN15 - POLR2J2 - FBXO24 - DNAJC2 - GNB2 - PILRB - PILRA - AGFG2 - MOGAT3 - DPY19L2P2 - GATS - C7orf52 - SLC26A5 - C7orf61 - UFSP1 - LRCH4 - ACHE - SPDYE3 - SPDYE2 - ORC5 - SERPINE1 - FIS1 - PCOLCE - ACTL6B - SRRT - PMS2P1 - POLR2J - ALKBH4 - POLR2J3 - ZCWPW1 - MEPCE - RELN - SLC12A9 - PSMC2 - MOSPD3 - GIGYF1 - RABL5 - TFR2 - TRIP6 - SPDYE6 - VGF - ZAN - PVRIG - PRKRIP1 - ORAI2 - TSC22D4 - TRIM56 - ARMC10 - PLOD3 - MYL10 - PMPCB" |
| chr7_102735276 | 26.7 | "RPL19P12 - UPK3BL - LOC100216545 - SPDYE2L - MIR4285 - RASA4 - LRRC17 - ZNHIT1 - SH2B2 - AP1S1 - EMID2 - CUX1 - LRWD1 - FAM185A - FBXL13 - NAPEPLD - CLDN15 - POLR2J2 - DNAJC2 - MOGAT3 - DPY19L2P2 - C7orf52 - SLC26A5 - LHFPL3 - SPDYE2 - ORC5 - SERPINE1 - FIS1 - POLR2J - ALKBH4 - POLR2J3 - MLL5 - RELN - PSMC2 - LOC645591 - RABL5 - LOC723809 - SPDYE6 - VGF - PRKRIP1 - ORAI2 - ARMC10 - PLOD3 - MYL10 - PMPCB" |
| chr8_454083 | 26.7 | "C8orf42 - ERICH1 - ZNF596 - CLN8 - FBXO25 - LOC286083 - OR4F21 - RPL23AP53 - MIR596 - MYOM2 - DLGAP2 - ARHGEF10 - KBTBD11" |
| chr9_39708311 | 26.7 | "FAM201A - RG9MTD3 - ALDH1B1 - FRMPD1 - ANKRD18A - ZNF658 - FAM75A7 - IGFBPL1 - FAM74A1 - ZNF658B - EXOSC3 - FAM75A2 - SHB - FAM75A1 - LOC653501 - FAM75A3 - FAM75A5 - FAM74A3 - DCAF10 - CNTNAP3 - MCART1" |
| chr10_47454088 | 26.7 | "FAM25B - FAM25G - LOC100133308 - MIR3156-1 - C10orf10 - ZNF488 - AGAP4 - FRMPD2 - CTSL1P2 - ANTXRL - MARCH8 - C10orf25 - LOC220980 - ALOX5 - ANXA8L2 - FAM21C - PTPN20B - GDF2 - GDF10 - LOC338579 - LOC399753 - BMS1P5 - FAM35B - FAM35B2 - PPYR1 - FAM21B - RBP3 - AGAP9 - LOC642826 - LOC643650 - FAM25C - PTPN20A - ANXA8 - BMS1P1 - ANXA8L1 - LOC728643 - FRMPD2P1 - ZNF22 - OR13A1 - SYT15 - RASSF4 - ANUBL1 - GPRIN2" |
| chr11_48651447 | 26.7 | "NR1H3 - CELF1 - C1QTNF4 - PTPMT1 - OR4X2 - OR4B1 - DDB2 - F2 - NUP160 - FNBP4 - FOLH1 - MTCH2 - OR4C3 - OR4S1 - OR4C13 - OR4C12 - PACSIN3 - OR4X1 - ARHGAP1 - FAM180B - OR4A47 - OR4C45 - LRP4 - LOC440040 - LOC441601 - MYBPC3 - NDUFS3 - ACP2 - KBTBD4 - PSMC3 - PTPRJ - RAPSN - LOC646813 - SPI1 - SNORD67 - C11orf49 - ZNF408 - AGBL2 - ARFGAP2 - MADD - SLC39A13 - ATG13 - CKAP5" |
| chr12_169304 | 26.7 | "LOC100271702 - LOC100288778 - LOC100292680 - FBXL14 - DCP1B - ERC1 - B4GALNT3 - IQSEC3 - NINJ2 - LOC574538 - RAD52 - KDM5A - WNK1 - SLC6A12 - SLC6A13 - LRTM2 - FAM138D - CACNA1C - ADIPOR2 - WNT5B - CCDC77 - CACNA2D4" |
| chr14_105735612 | 26.7 | "ZBTB42 - SIVA1 - AHNAK2 - CKB - TRMT61A - TDRD9 - C14orf79 - PLD4 - ADSSL1 - CRIP1 - CRIP2 - MGC23270 - EIF5 - AKT1 - PACS2 - PPP1R13B - NUDT14 - KIF26A - KIAA0284 - C14orf80 - BRF1 - GPR132 - NCRNA00226 - NCRNA00221 - JAG2 - ASPG - KLC1 - TMEM179 - C14orf180 - MIR203 - MARK3 - CDCA4 - INF2 - LOC647310 - SNORA28 - XRCC3 - ZFYVE21 - TMEM121 - C14orf153 - ADAM6 - BTBD6 - MTA1 - BAG5 - C14orf2 - KIAA0125" |
| chr15_27000239 | 26.7 | "SNORD116-1 - SNORD116-2 - SNORD116-3 - SNORD116-4 - SNORD116-5 - SNORD116-6 - SNORD116-7 - SNORD116-8 - SNORD116-9 - SNORD116-10 - SNORD116-11 - SNORD116-12 - SNORD116-13 - SNORD116-14 - SNORD116-15 - SNORD116-16 - SNORD116-17 - SNORD116-18 - SNORD116-20 - SNORD116-21 - SNORD116-22 - SNORD116-23 - SNORD116-24 - SNORD116-25 - SNORD115-2 - SNORD116-26 - SNORD116-27 - SNORD115-3 - SNORD115-4 - SNORD115-5 - SNORD115-6 - SNORD115-7 - SNORD115-8 - SNORD115-9 - SNORD115-10 - SNORD115-11 - SNORD115-12 - SNORD115-13 - SNORD115-14 - SNORD115-15 - SNORD115-16 - SNORD115-17 - SNORD115-18 - SNORD115-19 - SNORD115-20 - SNORD115-21 - SNORD115-22 - SNORD115-23 - SNORD115-25 - SNORD115-26 - SNORD115-29 - SNORD115-30 - SNORD115-31 - SNORD115-32 - SNORD115-33 - SNORD115-34 - SNORD115-35 - SNORD115-36 - SNORD115-37 - SNORD115-38 - SNORD115-39 - SNORD115-40 - SNORD115-41 - SNORD115-42 - SNORD115-43 - SNORD115-44 - SNORD116-28 - SNORD116-29 - SNORD115-48 - SNORD115-24 - HBII-52-27 - HBII-52-28 - HBII-52-45 - HBII-52-46 - GOLGA8F - PAR1 - GABRA5 - GABRB3 - GABRG3 - GOLGA8G - SNORD108 - SNORD109A - SNORD109B - SNORD115-1 - SNORD64 - PAR4 - PAR-SN - IPW - HERC2P9 - WHAMML2 - OCA2 - ATP10A - SNRPN - SNORD116-19 - UBE3A - PAR5 - HERC2 - SNURF - SNORD107" |
| chr16_32406646 | 26.7 | "KIAA0664L3 - MIR762 - BCKDK - ZNF267 - SRCAP - STX1B - ZNF689 - ZNF720 - COX6A2 - ZNF785 - ZNF688 - PRSS36 - CTF1 - ZNF48 - SEPHS2 - ZNF629 - TP53TG3 - FUS - PYDC1 - NCRNA00095 - ZNF843 - PYCARD - PRSS53 - ITGAD - ITGAL - ITGAM - ITGAX - SLC6A10P - LOC390705 - CSDAP1 - HERC2P4 - TRIM72 - AHSP - ZNF771 - PHKG2 - FBXL19 - PRSS8 - UBE2MP1 - FBRS - C16orf58 - SLC5A2 - ZNF747 - SNORA30 - STX4 - TGFB1I1 - TP53TG3B - PRR14 - VKORC1 - DCTPP1 - ZNF768 - ZNF668 - ARMC5 - HSD3B7 - MYST1 - C16orf93 - ZNF764 - BCL7C - ORAI3 - ZNF646 - SETD1A - RNF40" |
| chr17_21609936 | 26.7 | "KCNJ18 - MTRNR2L1 - AKAP10 - SLC47A2 - ALDH3A1 - USP22 - CDRT15L2 - C17orf103 - DHRS7B - FAM27L - FLJ36000 - LGALS9B - CCDC144NL - C17orf51 - CCDC144C - KCNJ12 - MAP2K3 - KRT16P3 - TMEM11 - SPECC1 - ULK2" |
| chr19_40509554 | 26.7 | "LOC100129935 - C19orf69 - PAK4 - CEACAM5 - RABAC1 - SPINT2 - DLL3 - CEACAM3 - CEACAM7 - CEACAM4 - HNRNPUL1 - MAP4K1 - EGLN2 - FBXO17 - RASGRP4 - CLC - EID2B - RINL - FBXO27 - C19orf47 - LYPD4 - HIPK4 - FAM98C - CAPN12 - LGALS16 - TTC9B - CYP2A6 - CYP2A7 - CYP2A13 - CYP2B6 - CYP2B7P1 - CYP2F1 - EID2 - ZNF780B - ECH1 - GGN - AKT2 - FBL - SIRT2 - SIPA1L3 - PLD3 - EIF3K - IL28A - IL28B - IL29 - ZNF780A - C19orf54 - GRIK5 - LGALS13 - CYP2S1 - SERTAD3 - SERTAD1 - HNRNPL - ZNF546 - NCCRP1 - SYCN - LEUTX - SELV - B3GNT8 - PAPL - LGALS4 - LGALS7 - SPRED3 - LGALS17A - MAP3K10 - CEACAM6 - ATP1A3 - NFKBIB - RAB4B - PAF1 - SARS2 - SAMD4B - ATP5SL - MED29 - AXL - LGALS14 - EXOSC5 - PSMC4 - PSMD8 - LRFN1 - PRX - SPTBN4 - CATSPERG - BCKDHA - MRPS12 - RPS16 - RPS19 - RYR1 - DMRTC2 - C19orf33 - TMEM91 - BLVRB - PLEKHG2 - LGALS7B - SNRPA - SUPT5H - MIR641 - TGFB1 - ZFP36 - ADCK4 - CNTD2 - ITPKC - B9D2 - ACTN4 - MIA - DPF1 - LTBP4 - FCGBP - CEACAM21 - CCDC97 - YIF1B - ARHGEF1 - DYRK1B - NUMBL - TIMM50 - SHKBP1 - KCNK6 - PPP1R14A - GMFG - CD79A" |
| chr19_47547007 | 26.7 | "SNAR-G1 - SNAR-A1 - SNAR-A2 - SNAR-A12 - BSPH1 - SNAR-A3 - SNAR-A5 - SNAR-A7 - SNAR-A11 - SNAR-A9 - SNAR-A4 - SNAR-A6 - SNAR-A8 - SNAR-A13 - SNAR-A10 - SNAR-C2 - SNAR-C4 - SNAR-E - SNAR-C5 - SNAR-C1 - SNAR-C3 - SNAR-G2 - SNAR-A14 - MIR3191 - MIR3190 - MIR320E - SAE1 - CGB - PPP1R13L - CD3EAP - RUVBL2 - KDELR1 - CLASRP - KPTN - CGB1 - CGB2 - LMTK3 - CKM - AP2S1 - NTN5 - CRX - PPM1N - KLC3 - DACT3 - SIX5 - IGFL2 - DBP - ZNF296 - ZNF114 - SPACA4 - DMPK - DMWD - EMP3 - ERCC1 - ERCC2 - CARD8 - ZC3H4 - FBXO46 - FOSB - SYNGR4 - PPP1R15A - EML2 - FTL - FUT1 - FUT2 - PRR24 - PRKD2 - CCDC9 - IRF2BP1 - FGF21 - GIPR - BBC3 - GPR77 - DHDH - GPR4 - NKPD1 - TPRX1 - MAMSTR - IZUMO1 - GRIN2D - GRLF1 - STRN4 - GYS1 - GLTSCR2 - GLTSCR1 - EHD2 - FOXA3 - MYPOP - NANOS2 - IGFL1 - KCNJ14 - BLOC1S3 - IGFL3 - LHB - LIG1 - MIR330 - IGFL4 - NOVA2 - NPAS1 - NUCB1 - HSD17B14 - QPCTL - FAM83E - RASIP1 - TMEM160 - PNMAL1 - TMEM143 - PPP5C - CABP5 - SPHK2 - MEIS3 - PTGIR - PNMAL2 - PLEKHA4 - MARK4 - BAX - BCAT2 - RPL18 - RTN2 - ELSPBP1 - SEPW1 - HIF3A - SLC1A5 - SEC1 - SLC8A2 - SNRPD2 - SULT2B1 - SULT2A1 - SNORD23 - MIR642A - C5AR1 - TULP2 - CCDC61 - VASP - MIR769 - CA11 - TRAPPC6A - FKRP - GEMIN7 - OPA3 - CALM3 - RSPH6A - SYMPK - GRWD1 - CCDC8 - ZNF541 - PLA2G4C - NAPA - PGLYRP1 - EXOC3L2 - CYTH2 - CCDC114 - DKFZp434J0226 - GNG8 - DHX34" |
| chr19_48486426 | 26.7 | "SNAR-G1 - SNAR-A1 - SNAR-A2 - SNAR-A12 - BSPH1 - SNAR-A3 - SNAR-A5 - SNAR-A7 - SNAR-A11 - SNAR-A9 - SNAR-A4 - SNAR-A6 - SNAR-A8 - SNAR-A13 - SNAR-A10 - SNAR-C2 - SNAR-C4 - SNAR-E - SNAR-C5 - SNAR-C1 - SNAR-C3 - SNAR-G2 - SNAR-A14 - MIR3191 - MIR3190 - MIR320E - MIR4324 - SAE1 - CGB - RUVBL2 - KDELR1 - KPTN - PNKP - PTH2 - SIGLEC11 - CGB1 - CGB2 - LMTK3 - AP2S1 - CPT1C - ALDH16A1 - NTN5 - CRX - CCDC155 - DACT3 - IGFL2 - AP2A1 - DBP - ZNF114 - SPACA4 - C19orf76 - EMP3 - FCGRT - ATF5 - CARD8 - ZC3H4 - FLT3LG - RPL13A - SYNGR4 - NUP62 - PPP1R15A - FTL - FUT1 - FUT2 - PRR24 - PRKD2 - IL4I1 - CCDC9 - FGF21 - SNORD35A - SNORD34 - SNORD33 - SNORD32A - BBC3 - DKKL1 - GPR77 - DHDH - TPRX1 - MAMSTR - IZUMO1 - SLC6A16 - GRIN2D - GRLF1 - STRN4 - GYS1 - GLTSCR2 - GLTSCR1 - EHD2 - HRC - PRMT1 - IRF3 - KCNA7 - IGFL1 - KCNJ14 - IGFL3 - LHB - LIG1 - SIGLEC16 - MIR150 - IGFL4 - NPAS1 - NTF4 - NUCB1 - NOSIP - HSD17B14 - VRK3 - PTOV1 - TRPM4 - FAM83E - RASIP1 - TMEM160 - PIH1D1 - C19orf73 - PNMAL1 - TMEM143 - PPP5C - CABP5 - PRRG2 - SPHK2 - MEIS3 - SLC17A7 - RCN3 - PTGIR - PNMAL2 - PRR12 - PLEKHA4 - BAX - SCAF1 - BCAT2 - TSKS - RPL18 - RPS11 - RRAS - ELSPBP1 - LIN7B - SEPW1 - HIF3A - SLC1A5 - SEC1 - SLC8A2 - SNRNP70 - SULT2B1 - SULT2A1 - SNORD23 - C5AR1 - RPL13AP5 - TULP2 - CCDC61 - MIR769 - CA11 - FKRP - TBC1D17 - FUZ - CALM3 - MED25 - BCL2L12 - GRWD1 - CCDC8 - ZNF541 - AKT1S1 - SNORD35B - TEAD2 - PPFIA3 - PLA2G4C - NAPA - PGLYRP1 - CYTH2 - CCDC114 - DKFZp434J0226 - CGB5 - CGB7 - CGB8 - GNG8 - CD37 - DHX34" |
| chr21_46676181 | 26.7 | "ADARB1 - FTCD - HSF2BP - C21orf84 - C21orf90 - MCM3AP-AS1 - COL6A1 - COL6A2 - CSTB - SIK1 - RRP1B - DIP2A - POFUT2 - ICOSLG - C21orf125 - LOC284837 - DNMT3L - AIRE - PRMT2 - KRTAP12-2 - KRTAP12-1 - KRTAP10-10 - ITGB2 - NCRNA00162 - NCRNA00175 - KRTAP10-4 - KRTAP10-6 - KRTAP10-7 - KRTAP10-9 - KRTAP10-1 - KRTAP10-11 - KRTAP10-2 - KRTAP10-5 - KRTAP10-8 - KRTAP10-3 - KRTAP12-3 - KRTAP12-4 - KRTAP10-12 - LSS - PCNT - PFKL - PCBP3 - C21orf58 - YBEY - TSPEAR - AGPAT3 - PWP2 - S100B - LOC642852 - SLC19A1 - SUMO3 - TRAPPC10 - TRPM2 - NCRNA00163 - C21orf122 - UBE2G2 - PTTG1IP - C21orf2 - COL18A1 - LRRC3 - C21orf33 - C21orf56 - C21orf67 - C21orf70 - PDXK - RRP1 - MCM3AP" |
